# On Man, and on the Development of His Faculties, &c.

**Published:** 1837-01

**Authors:** 


### ART. II.

*Sur l'Homme et le Developpement de ses Facultés; ou, Essai de Physique Sociale.* Par A. QUETELET, Sécretaire Perpetuel de l'Académie Royale de Bruxelles, &c.—Deux Tomes. 8vo. *Paris*, 1835.

*On Man, and on the Development of his Faculties; or, an Essay on Social Physics.* By A. QUETELET, &c.

*Life Tables, founded on the Discovery of a Numerical Law, &c. Illustrated by a new Theory of the Causes producing Health and Longevity.* By T. R. EDMONDS, B.A.—1832.

*Tables shewing the total Number of Persons assured in the Equitable Society from its commencement in September,* 1762, *to January* 1*st*, 1829, &c.—1834.

THE introduction and use of accurate measures form an epoch in the history of all sciences; dividing a period of obscurity, where doubt and dogmatism are perpetually at war, from a brighter period of development, in which every phenomenon is successively reduced to its natural relations. Astronomy, natural philosophy, chemistry, and meteorology, by the gradual invention of instruments, shook off the delusions of astrology, alchemy, and other sciences falsely so called; and, by the application of calculation, itself only an instrument for indirectly determining quantities and relations, they are every day attaining a further and surer possession of the laws of the universe.

The employment in medicine of the mathematics, which have been employed so advantageously in other sciences, is, in a certain sense, impracticable: to pursue the elements of the human body through the cycle of organization, and to deduce all their possible combinations and manifestations from their simple properties, is a task too high for the present imperfect state of mathematical analysis, as well as for the more imperfect means of observation yet known. It is not, however, as some suppose, because matter organized and living is subject to no law, but because its changes are difficult to seize, and infinitely complex, that the mathematician encounters insuperable obstacles in its investigation; for, in chemistry and meteorology, when dealing with inorganic atoms, similar difficulties occur: the movements of the atmosphere have not yet been expressed by a general law, "yet the curve described by a single molecule of air or vapour is regulated as certainly as the planetary orbits; the only difference is that caused by our ignorance."* It is an easy matter to predict when the earth will again occupy its present position; to foresee the courses the planets will follow; to calculate even the unseen orbits of the comets; but who can tell, *a priori*, where a passing cloud will be borne; what form an organic substance will assume; much less, what the life and actions of a human being will be? Nearly all the events on which

* Laplace.

our judgment is daily exercised, the results of our actions, and the duration of our very existence, are, considered in an isolated state, uncertain: they may terminate in several ways; but experience has established that all general facts have different probabilities of happening, and, whether the probabilities can or cannot be exactly measured, men are compelled to resolve and act on them. Observation, too, has shewn that physiological acts, when examined and measured in a great number of individuals, furnish a mean result deviating no more from a fixed standard than other physical phenomena. Statistical enumerations prove that actions which spring from hidden principles in the organization, and from accidents to which it is exposed, are constant, or constantly modified to a determinable extent. In France, the mean annual number ot deaths, in the six years 1826—31, was 813,329; the maximum was 837,145, the minimum 791,125: the mean annual number of murders committed was 234, and the deviation in the whole period never exceeded one-eighth: in the five years, 1827—31, the yearly number of *accidental* deaths was 4,834, or one-thirteenth more or less. On the concurrence of how many tumultuous passions, wayward thoughts, and states in man, and of what multiplied circumstances, did each death, murder, and fatal accident depend! Yet so constant were the causes in operation, as every year to produce a result fluctuating less than the mean temperature of the atmosphere or the products of the soil.

Mankind, then, studied in large numbers, possess certain common qualities, which remain the same or vary according to determinable principles. Equal numbers of the same race have the same weight at birth, grow in the same ratio, live to the same mean age, die in the same proportion when exposed to similar causes: their nutritive, procreative, muscular, perceptive, and spiritual powers are identical, and are expressed in acts of equal frequency and duration, or differ according to ratios which science may hope to determine. The enumeration and generalization of these facts constitute what Laplace happily named "Vital Statistics."

Gioja divided statistics into six departments, each of which is ably discussed in the "Filosofia di Statistica." That relating to population, and the laws of its renewal, development, decline, and disorganization, comprehends the same class of facts as vital or medical statistics; and vital statistics are indisputably a part of general physiology; they form part of our medical studies; yet the great questions of population, mortality, and national health, which have now forced themselves on the attention of governments, and of every educated person, owe little of their progress to the medical profession. Professor Casper and Dr. Bisset Hawkins have the merit of writing distinct treatises on the subject; but we are compelled to admit that the work of M. Quetelet evinces a better knowledge of principles, and contains more original facts, than either of the medical treatises. In the present article, however, restricted to the examination of human mortality, little use can be made of anything but the facts recorded in M. Quetelet's volumes. Although a distinguished mathematician, he is not well acquainted with the true principles of life admeasurement; which, contrary to popular belief, are nowhere so well understood as in England. Sweden was the first country in which returns were published of the living and the dying at different ages in the whole kingdom, for several years in the last century. In England, a census of the population was made in 1801, and has since been repeated every ten years; the parish registers of burials and deaths have been published; and, in 1821, the ages of nine-tenths of the population were determined. In France, the registration of the total number of births and deaths is complete, but no attempt has been made since 1789 to ascertain the ages of the living. The relative mortality of the French nation is unknown: the table of Duvillard, published in the "Annuaire," is of no more value than the exploded Northampton Table. In Belgium, an enumeration of the ages of the living was effected in 1829; but M. Quetelet appears not to have availed himself of this enumeration in the construction of the tables of mortality published in his present work. The ages, but not the deaths, of the inhabitants of the United States have been published. So, notwithstanding the capital and inexcusable error of neglecting the enumeration of the ages in the last census,* no country, except Sweden, possesses more valuable statistical materials than England; and the writings of Price, Morgan, Milne, Gompertz, and Edmonds, exhibit clearer theoretical views of the laws of mortality than can be found in the works of any modern foreign writer. Halley published the first table of mortality in the Philosophical Transactions, (1693;) Kerrseboom, a Dutch writer, (1733-42,) and Deparcieux, (1746,) a Frenchman, formed tables of mortality on correct principles from the lives of annuitants; and Lambert, a German, first demonstrated (1772) the general properties of these tables mathematically: so that the several European nations have each contributed something to the science of human mortality.

We now proceed to examine the best means of life-admeasurements, and to investigate the mortality in various circumstances, without at present enquiring by what particular forms of disease death is brought about, or how it may be averted.

That at all times of his changeful existence man is liable to death, and that in infancy and advancing age vitality holds a less firm tenure of its clay than in youth and manhood, long experience has made an article of popular belief. But how observe and measure the force of this mortality? or, rather, how measure the

* Drs. Price, Morgan, Milne, in the Supplement to the Encyclopædia Britannica, and others, had pointed out the branches of enquiry which a census should embrace.

development and decrement, the friction and loss of that life which still carries on its own operations to their natural close? It is impossible to accomplish this by the consideration of one individual, as indeed it would be impossible to find out the pressure and temperature of the atmosphere with a single globule of mercury; but, by taking masses of mankind for observation, all the elements required for accurate admeasurement will be at once possessed. Where circumstances remain the same, an equal number die in a unit of time out of a certain number living; allowing us to infer that the force of death and disorganization is equally distributed through time and space, and that, if there is any apparent irregularity, it arises from the small extent of the observation, unless some cause of perturbation has interfered. In an urn filled with black and white balls, in the proportion of one hundred black to one thousand white ones, the balls would probably not be drawn in this proportion, if only eleven or 110 were drawn; but, by going on, it would be found, on placing them in another urn, that the proportions in the end would be the same in both, and that they had been drawn in the proportion of one black to ten white balls. So, if one hundred men were singled out, each twenty or thirty years old, one or two, or none of them, may die in the year; but, if one thousand men were selected, one in every hundred would most likely die, and the probability of this proportion would augment as the operation was extended. To determine the mortality, then, it is only necessary to have observations sufficient to ensure uniformity, and to compare the two quantities, the living and the dying, the relations of which in a given time express the law of mortality, or rather of vitality; for, considered in any way, life always prevails over death with a power represented by the mass and number living.

If the population of England was fourteen millions, and the deaths in a year three hundred thousand, the absolute annual mortality would be expressed by $\frac{300000}{14000000}$: the mortality, then, may always be expressed by the number of deaths out of a certain number of individuals *living* a given *time:* the law of mortality expresses the relation of the deaths to the *lifetime.* For its determination, the living, the dying, the time, must be observed, and may be looked upon as three different but essential elements, two of which remaining invariable, the range of the third measures the force of mortality. Let the living be a million, the dying one, and the number living be constantly maintained the same by the regular accession of a new life in lieu of that expiring; then the interval of *time* between each death will indicate the force of mortality. If, in the last century, a million Swedish children, between birth and five years of age, could have been brought at once before the eye, and observed, one would have been seen expiring every sixth minute. Sometimes several would fall together, at other times there would be a longer pause; but the mean interval would be six minutes. If, instead of children, the living were youths between the ages of ten and fifteen, the period of man's greatest potential if not actual productive life, one would die every eighty-fifth minute; and from this culminating point, where the strokes of death beat slowly, they would quicken as the ages of the observed million advanced, till at last, on the utmost limit of human longevity, one would fall every minute. The different rate of mortality at different ages, and in different populations, may thus be compared:

*Mean Rate of Dying in Minutes, out of a Million living at each Age.*

| Between Ages | Sweden, 1776-95. | Belgium, 1829. | England, 1818-24. |
|---|---|---|---|
| 0 and 5 | One death every 6th* | 7.99th | 11.53d minute. |
| 5 ... 10 | ... 39 | 60.46 | 80.92 ... |
| 10 ... 15 | ... 85 | 97.40 | 99.25 ... |
| 15 ... 20 | ... 75 | 79.51 | 69.21 ... |
| 20 .. 30 | ... 59 | 57.80 | 52.55 ... |
| 30 ... 40 | ... 45 | 52.06 | 43.48 ... |
| 40 ... 50 | ... 33 | 38.68 | 35.54 ... |
| 50 . 60 | ... 22 | 24.24 | 25.29 ... |
| 60 ... 70 | ... 11 | 13.66 | 13.08 ... |
| 70 ... 80 | ... 5 | 5.78 | 5.54 ... |
| 80 ... 90 | ... 2½ | 2.94 | 2.47 ... |
| 90 .. 100 | ... 1 | 1.72 | 1.43 ... |

In Sweden, out of a million children living, one died every six minutes; in Belgium, one in every eight minutes; in England, only one in every eleven and a half minutes.

Instead of the *number living* remaining invariable, if the *time* be fixed (at one year), and the *dying* be one, as in the former instance, the *living* express the relative mortality.

*Mean Numbers living, out of which one Death takes place annually.*

| Between Ages | Sweden. | Belgium. | England. |
|---|---|---|---|
| 0 and 5 | 1 death in 12 living. | 1 death in 15 living. | 1 death in 22 living. |
| 5 ... 10 | ... 74 ... | ... 115 ... | ... 154 ... |
| 10 ... 15 | ... 177 ... | ... 185 ... | ... 189 ... |
| 15 ... 20 | ... 143 ... | ... 151 ... | .. 135 ... |
| 20 ... 30 | ... 112 ... | ... 110 ... | ... 100 ... |
| 30 ... 40 | ... 86 ... | ... 100 ... | ... 83 ... |
| 40 .. 50 | ... 60 ... | ... 73 ... | ... 68 ... |
| 50 ... 60 | ... 42 ... | ... 46 ... | ... 48 ... |
| 60 ... 70 | ... 20 ... | ... 26 ... | ... 25 ... |
| 70 ... 80 | ... 10 ... | ... 11 ... | ... 11 ... |
| 80 ... 90 | ... 5 ... | ... 6 ... | ... 5 ... |
| 90 ... 100 | ... 3 ... | ... 3 ... | ... 3 ... |

The differences here are very striking: the numbers result immediately from the division of the living by the annual deaths; no decimals need in general be employed, which makes it plainer for the general reader; and the number living at all ages, out of which one death takes place annually, answers to the mean duration of life; so that, if one death in thirty-seven occurs yearly, the mean age attained by the entire population is thirty-seven years. Wherefore some prefer this to the following method, in which the

* For Sweden, fractions have been omitted.

living (1000,) and the time (a year,) are fixed, while the *dying* mark the scale of mortality.

*The Number of Deaths which occur annually out of a Thousand Persons living.**

| Between Ages | Sweden, 1776-95. | Belgium, 1829. | England and Wales, 1818-24. |
|---|---|---|---|
| 0 and 5 | 85.0 | 65.8 | 45.6 |
| 5 ... 10 | 13.6 | 8.7 | 6.5 |
| 10 ... 15 | 6.2 | 5.4 | 5.3 |
| 15 ... 20 | 7.0 | 6.6 | 7.6 |
| 20 ... 30 | 8.9 | 9.1 | 10.1 |
| 30 ... 40 | 11.6 | 10.0 | 12.1 |
| 40 ... 50 | 16.1 | 13.6 | 14.8 |
| 50 ... 60 | 23.9 | 21.7 | 20.8 |
| 60 ... 70 | 49.3 | 38.5 | 40.2 |
| 70 ... 80 | 104.1 | 90.9 | 94.9 |
| 80 ... 90 | 197.4 | 178.8 | 212.7 |
| 90 ...100 | 351.3 | 304.7 | 367.8 |
| All ages, | 26.8 | 22.7 | 20.3 |

The numbers living and dying in these scales may readily be converted into mass or weight, and the mortality may be determined by weighing instead of counting the population; for, the average weight of a boy seven years old, being 43 lbs., of a man aged forty-five years, 140 lbs., a thousand of the former would weigh 43,000 lbs., while a thousand of the latter would be equivalent to a mass of 140,000 lbs. avoirdupois, and the mass eliminated, disorganized, dying from the boys, would be 310 lbs., from the men 745 lbs., without at all altering the law of mortality; inasmuch as the relations of the mass living and dying would remain precisely the same as the relations of the numbers. Whether the numbers, or mass, or muscular or intellectual force of a nation were regarded, the relations of the existing and perishing would be expressed by the same law.

In examining the vitality of different ages, a very extensive range in the scale of intensity is perceived: man is never equally mortal during two summers of his life; and, as his being revolves through its course in minutes, years, or equal intervals, the vitality at first increases, and then decreases in geometrical progression; but, as experience has shewn, the rate of progression changes. There are three orders of progression. The first extends from birth to the second teething, when the body becomes every year less liable to death; the second prevails from puberty over manhood, when the force of mortality slowly creeps on, and grows stronger; the third sets in on the eve of old age, and with a more rapidly accumulating energy. Dr. Price pointed out these remarkable periods;† but to Mr. Edmonds the honour is due of expressing their rates of diminishing or increasing mortality in the subjoined numbers.

* From a paper by Mr. Edmonds, in the British Medical Almanack for 1836.

† Dr. Price's works by W. Morgan, vol. ii., p. 222. Till the age of five, human life, like a fire beginning to burn, is very feeble, &c. The constants may be a little too high or too low.

*Numerical Values, which indicate the rate of Increase or Decrease of the Force of Mortality, in a given Time, assumed to be one Year.*

| Constants. | | Period over which constant presides. |
|---|---|---|
| .676 | . . | Infancy (from birth to eight years of age.) |
| 1.030 | . . | Manhood (from twelve to fifty-five years of age.) |
| 1.080 | . . | Old age (from fifty-five to end of life.) |

By means of the three constants, it is easy, when the mortality of one year is known, to deduce from it that of the next; for example, if the mean mortality at the age of twenty-five be 1 per cent., the mortality in the next year (twenty-six) will be nearly 1.03, which, multiplied by the constant of the period, gives the mortality for the twenty-seventh year, &c. up to fifty or sixty; where the second merges into the third period, whose constant must then be employed. As the rate of mortality is ordinarily obtained for equal periods of ten years, it may be well to bear in mind that the mortality at the ages between twenty and fifty increases one-third every ten years: in old age it should nearly double.

Man owes nature a debt, for which he pays compound interest; the constants shew how the rate of interest varies.

The deaths in the first months after birth are, from the rude shocks to which young life is exposed, higher than the rate of infancy indicates; yet there is undoubtedly a law that would extend even to conception, and the expression of that law would perhaps differ little from the constant of infancy. The rates of dying in infancy and manhood resolve gradually one into the other; so that, probably, when the mortality is low in childhood its constant terminates at six or seven years of age; when high, at ten or twelve; and often, towards puberty, the rate of mortality seems stationary. Whether the period of manhood is in direct or inverse proportion to the extent of the period preceding, remains yet an unsolved but very interesting problem; the point of separation between manhood and old age ranges from the fiftieth to the sixtieth year in different classes of the population.

Infancy, manhood, and old age are, then, three essentially distinct periods of human life; and must not be confounded with the arbitrary and subordinate, but useful divisions of some physiologists. The ages of growth, generation, and decay have, in fact, from the time of Aristotle, attracted the attention which their paramount importance demands; nor in pathology is any other division of life so useful at the present day, either to guide the practitioner in the investigation of disease or in prognosis; yet practical writers appear no more aware than the ancients that the number of deaths at each period of life happens according to a predestined order.

A similar law of mortality presides over all organized nature, although physiologists have yet made no observations by which it can be numerically expressed. The embryo plant, the bud, the flower, grows every day less liable to death by internal debility or external injury; before and during florescence, its existence is most flourishing; then follows old age, and life,—blossoms, leaves, and stem,—droop and decay. As millions of flowers, and untimely fruits, and wild seeds, and young plants, perish for one that is lost at a later time, so the opening eggs of insects, and the worm, the spawn of fishes and their fry, the young of birds, and the seed and offspring of all mammiferous animals, are exposed to daily risks; but acquire vital tenacity and force, till, their age of puberty and reproduction having passed over, they successively perish in old age, according to an accelerating progression. On the eve of reproduction, the human species, all quadrupeds, the birds of the air, the inhabitants of the seas, and all tribes of vegetables, have the widest prospect of life; in this spring of their existence, they are beautiful and strong; the past is short, the future is long; and man, who looks before, is full of hope. When physiologists have studied organization as attentively in time as they have in space, it will be possible to deduce the general course of vital duration and decay; for all forms of life are subject to the laws of mortality; and by them the immemorial forests themselves are renewed through successive centuries.

Applied to measure, the influence of age on human mortality, our method has shewn that this ranges on the scale one thousand from 5 to 611: no other ordinary influence is so powerful; none which we are about to examine so important. The mortality of the two sexes differs in favour of females. It is well known that for fifteen girls, sixteen boys are born. To compensate for this, the mortality of male infants is greater than that of females. On an average, one child in twenty-two is still-born (in illegitimate children the proportion is two in twenty-four); and in West Flanders, according to M. Quetelet, the proportion of still-born males to still-born females was as fourteen to ten; it was the same in Berlin. The Swedish Tables, in which the sexes are distinguished, make the mortality of males greater than that of females all through life; and this accords with several other observations. In the eighteen years, 1813-30, the mortality, according to the English returns, was greater among males than females up to puberty and after fifty; during the period of childbearing, more females died than males.

Out of 1000 of each sex living in each interval of age, there died annually:

| Between Ages. | Males. | Females. |
|---|---|---|
| 0 — 5 | 53.5 | 46.0 |
| 20 — 30 | 10.1 | 10.4 |
| 30 — 40 | 11.4 | 12.4 |
| 40 — 50 | 14.9 | 14.9 |
| 60 — 70 | 45.3 | 41.2 |

The observations made in Belgium present nearly similar results, according to M. Quetelet's work. From the English Annuitants, (1785-1825), Mr. Finlaison has calculated tables which make the life of females five years and a half longer than the life of males: no physiologist can believe in the reality of this preposterous difference between male and female life; when the facts are laid before the public, it will probably be found that the well-known influence of selection interfered.

For comparing the relative mortality of different races and nations, few observations to be depended upon exist. Sir F. D'Ivernois, Mr. Rickman, Moreau de Jonnes, Dr. Bisset Hawkins, and M. Quetelet, however, have calculated that the absolute annual mortality of the Russians was, within the last twenty years, one in 27; the Prussians 1 in 36.2; the French, 1 in 39.7; the Dutch, 1 in 38; the Belgians, 1 in 43.1; the English, 1 in 51;* the Sicilians, 1 in 32; the Greeks, 1 in 30; the inhabitants of Batavia, 1 in 26; of Iceland, 1 in 30. Admitting that the data from which these numbers have been derived are correct, the conclusions usually deduced from them are false. To obtain the absolute mortality, the population at all ages is employed. Now, we have seen that the mortality of infants and old people differs enormously from that of men in the bloom of life: where individuals of different ages are therefore mixed, from any cause, in differing proportions, the result on the scale of absolute mortality varies without indicating a change of intensity; as mercury may dilate less than spirits of wine in the tube of a thermometer, and yet mark the same temperature. To obtain the absolute vitality and mean duration of a race, at one or more times of its history, a generation should be followed from birth to death; and, where this cannot be done, the problem is to obtain a near approximation to such a case.

Among the agents which keep up, and supply the consumption of life, the most essential are animal and vegetable matter, water, and oxygen; as vitality "exists on many a thousand grains that issue out of dust," it is maintained by the unceasing decomposition of aliment; it ceases when carbonic acid and aqueous vapour are no longer exhaled. Food, water, air, fire, (our bodily elements,) are the limits of population; the effects of their entire withdrawal are well known; the effects of different gradations of privation may be traced from the horrors of the Black Hole in Calcutta, or the faces of frenzied hunger on the Medusa, through prisons and dungeons, to the huts and garrets of the poor, all over the world. Of the direct effects of privation and saturation on length of life, no scale has yet been formed; but excess joined with indolence, stimulants without action, are no less pernicious, in all likelihood, than the contrary condition. Hippocrates was undoubtedly justified in contending that health is best maintained by preserving an exact proportion between food and labour; or, rather, by keeping the mean supply, though somewhat irregular, equal to the mean consumption. Mr. Edmonds says he has ascertained that the English peerage are

* Given by M. Quetelet from an early calculation of Mr. Rickman; the corrected mortality is 1 in 43.7, according to Mr. Edmonds.

LIBRARY OF THE

Orleans Parish Medical Society subject to a high degree of mortality; this is partly owing to the number of delicate children reared, by which hereditary debility is propagated.

The effect of any external influence on vitality may be determined by either of the three classes, children, adults, or old men; and, the range on the scale being constantly greater when children are employed, they may be said to form a more *sensible instrument* of admeasurement than young men in the prime of life. But children cannot be used to determine the influence of change of climate; they remain in their birth-place; while manhood, fraught with intellect, enterprise, health, and physical independence, embodying a people's strength, alone furnishes its disposable force, extending without, under unaccustomed skies. Young men are the first merchants, missionaries, and colonists; they constitute the military force of a country. The natural resistance to their extension over the globe is shewn by the sickness and mortality of the British troops in different climates: the resources of the country by which these difficulties are met are equally apparent.

*Annual Rate of Mortality per* 1000.

| | |
|---|---|
| Ireland, . . . . . . . . . . . . . . | 15. |
| Ionian Islands, . . . . . . . . . . . | 26. |
| Bengal, . . . . . . . . . . . . . . | 57. |
| West Indian Islands (Windward and Leeward), | 113. |
| Jamaica and Honduras . . . . . . . . | 155. |

The mortality of the English soldier increases as he removes from the climate of his race, birth, and childhood; till at last it becomes terrible: at the age of twenty-six and thirty he is as open and liable to death in the West Indies as the old man bent with years at home.

Independently of miasmatic emanations, and other causes everywhere fatal, it is erroneous to suppose, that any climate within the temperature man can sustain is to the above extent absolutely insalubrious. A hot climate is insalubrious for a race indigenous in a temperate or cold country, particularly when carrying with them the habits resulting from the exigencies of their native climate; but to its own autocthonous race it may be a nursing place of millions. Such is the state of things in India. The climate of the Madras Presidency, so fatal to the English soldier, is found salubrious by the Native troops; who, in their turn, would most likely perish in the cold changeful embrace of the English atmosphere.

The higher mortality of the Swedes is directly or indirectly due to their cold climate; the weaklier lives eliminated in infancy leave fewer but healthier men for the period of manhood than now exist in England or Belgium.

*Rate of Mortality in* 1000 *living at each age.*

| Between Ages | England, 1818-24. | Belgium, 1829. | Sweden, 1776-95. | Stockholm. |
|---|---|---|---|---|
| 0 and 5 | 45.6 | 65.8 | 85.0 | 216.2 |
| 20 ... 30 | 10.1 | 9.1 | 8.9 | 18.6 |
| 60 ... 70 | 40.2 | 38.5 | 49.3 | 69.1 |

This brings us to one of the complicated influences which, acting on all ages, has a very powerful effect on the absolute vitality; namely, residence in cities. The frequent plagues of past centuries no longer desolate our cities, although they yet hover, at Constantinople and Cairo, on the borders of Europe; smallpox has grown much less frequent; and cholera alone has passed the *cordon sanitaire* which the precautions of modern hygiène have drawn around this country. Still the mortality in cities is high, and merits careful consideration.

The mortality of cities and towns in Belgium is to the mortality of the country population, nearly as 4 to 3.

| | Population. | Mean number of deaths. | Deaths in 1000. |
|---|---|---|---|
| Cities and Towns | 998,118 | 270,26 | 27.1 |
| Country . . | 3066,091 | 652,65 | 21.3 |

The great mortality of childhood in Stockholm, compared with all Sweden, is illustrated above; the absolute mortality presents results equally unfavorable to residence in cities.

| | | Deaths. | | Deaths. |
|---|---|---|---|---|
| Stockholm;* | Males, | 1 in 17 | All Sweden, Males, | 1 in 33½ |
| | Females, | 1 in 21 | Females, | 1 in 36. |

The mortality of six English cities, York, Norwich, Plymouth, Hull, Portsmouth, and Liverpool, has been investigated by Mr. Edmonds;† the proportion of males deceased is made rather too high, as the military and maritime population was not included in the enumeration of the living, although the deaths were registered. The absolute mortality of males stated for the cities is founded on the assumption that the registered deaths are to be increased twenty per cent.

*Annual Mortality per* 1000 *in England and in six Cities.*

MALES.

| Ages . . . . 0—5 | 5—10 | 10—15 | 15—30 | 30—60 | Above 60 | All ages |
|---|---|---|---|---|---|---|
| England and Wales 53.0 | 7.2 | 4.9 | 8.8 | 15.3 | 77.7 | 21.5 |
| Cities . . . . 91.4 | 10.8 | 6.2 | 15.2 | 23.2 | 98.0 | 33.6 |

FEMALES.

| Ages . . . . 0—5 | 5—10 | 10—15 | 15—30 | 30—60 | Above 60 | All ages |
|---|---|---|---|---|---|---|
| England and Wales 45.6 | 6.6 | 5.2 | 9.3 | 15.2 | 75.3 | 20.5 |
| Cities . . . . 80.0 | 9.6 | 5.3 | 10.1 | 18.5 | 85.6 | 26.6 |

Looking at the absolute mortality alone, the greater number of persons living in towns, between the ages of fifteen and sixty, ought to render this low, as was well remarked by Dr. Price, who dwelt with emphatic earnestness on the fatality of cities, which he maintained "operated in checking population, and preventing the increase

CHARITY HOSPITAL,
MEDICAL LIBRARY.

* Dr. Price on Annuities, by Morgan. Vol. ii., p. 233-7.
† Lancet, December 12, 1835.

of mankind." Mr. Edmonds has ingeniously proved that the mortality of children in London has progressively diminished for the last century; but no practitioner who has had much experience among the poor will be surprised to find that still twice as many children perish in London as in the country.

They are chiefly young persons from the country, between the ages of fifteen and twenty-five, when emigration into cities is greatest, that furnish the Paris hospitals with cases of typhus fever: out of 133 patients observed by Louis, only four were born in Paris.* Twenty-eight out of sixty-three patients who had been at Paris less than ten months, died; while, of fifty-six who had lived there longer, only sixteen were lost. A city is a foreign climate to a countryman.

The method of life-admeasurement we have been examining admits of innumerable applications; and, notwithstanding its extreme simplicity, is easily misunderstood and misapplied. A few errors, common and obvious, but supported by the authority of respectable writers, will shew the present state of popular knowledge on the subject, and may serve as beacons to the younger student.

In estimating the influence of any cause which can but slightly affect vitality, the great disturbing influences must not be allowed to interfere: for instance, in examining the effect of two climates, or external conditions, little differing from each other, individuals of the same age, sex, riches, and residence, whether in cities or the country, should be compared. In passages like the following, this is lost sight of, where the mortality of men in the prime of life and this country is pronounced low, when less than that of men, women, infants, and old people in the city of Rome. "So great was the care taken of prisoners of war in this country, that, in the year 1813, the mortality amongst them was only one in fifty-five; not one half of what occurs to the whole population of Rome, although these persons were labouring under most of the privations which embitter or enfeeble existence!"†

In the *Précis d'Anatomie Pathologique* M. Andral states, (t. i., p. 428,) "that at the age of four or five years, three-fourths of the children who perish, die of tuberculous affections, &c. *At this age, therefore, more than in the years preceding, all irritation, every congestion is to be feared, &c.*" The practical directions following this supposition are entirely erroneous; the relative frequency of tuberculous deposits in the dead body increases from birth to the fourth or fifth year:‡ but irritation, congestion, and all fatal diseases become less frequent and dangerous from birth to the second teething; nor has any exception to this law ever been observed. It is unnecessary to give further examples of the practical errors into which distinguished medical writers have been induced, by want of

* Louis, Recherches sur la Fièvre Typhoide.

† Medical Statistics, p. 160. ‡ Dr. Clark on Phthisis.

acquaintance with the general law according to which human life wastes. M. Louis appears to have progressively invented his numerical method, and has necessarily fallen into some errors, from which a little research would have kept him free; for the "méthode numérique" is only a particular but most important application of principles well known in this country, at least since the time of Dr. Price, and explained a century ago by Deparcieux in France. M. Louis was evidently at first unacquainted with the law of mortality; but his applications of the numerical method were rendered accurate by the severely scientific character of his mind. He is the first who applied numbers with success in practical medicine.

Facts and figures were never more justly appreciated by the legislature of this country than in the present day: orators make their speeches persuasive by force of arithmetic; never were so many statistical returns procured; yet the elementary principles of statistics are far from being so extensively diffused in higher quarters as is desirable. Lord Brougham, in his last speech on Education,* entered into an elaborate calculation relative to the extent of instruction in different parts of England; assuming that the proportion of children was the same in London and Lancashire as in the rest of England; and, on this assumption, contradicted by calculations made in the last century, and by the enumeration of 1821, a weighty argument and a legislative enactment were urged. It is remarkable that no noble lord put his lordship right on a fact so familiar.

M. Villermé, from a comparison of the deaths at different ages in the marshy departments of France, lately announced "as a fact hitherto unknown, that the fatal influence of marshes is chiefly felt by young children." To confirm this statement, he divided 10,000 deaths in the manufacturing districts, and the same number in the agricultural counties of England, into those who died under, and those who survived their tenth year, but died before the fortieth; and, finding that the proportion of children buried in the marshy and manufacturing districts preponderated, inferred that the liability to death among children was increased to the same extent. All these calculations, founded on the *deaths alone,* admit by hypothesis that the proportion of children to the rest of the population *living* during the *time* the *deaths* happened, was the same in all the places compared. Here this was not the case; for the children living in the Island of Ely and the increasing manufacturing districts are more numerous than in the agricultural counties; so if the deaths were more numerous, the *living* were also more numerous, and the relation does not indicate a mortality so great as M. Villermé's method implies. Such is an example of the more common errors of writers in the "Annales d'Hygiène;" it is, however, only just to observe that M. Villermé was probably misled by the official tables

* Speech of Lord Brougham in the House of Lords, May 21, 1833. Ridgway.

in the third volume of the English Population Returns for 1831; where, in defiance of every principle and all authority, tables of mortality for each county were professedly deduced from the burials of a rapidly increasing population, and the proportions living, although known, were entirely overlooked. These tables assume that the proportion of children and young men was the same in the last century as in 1831, although the number of registered baptisms was only 237,029 in 1801, and 382,060 in the year 1830; increasing so rapidly as, in the course of thirty years, to raise the total population of England from about eight to fourteen millions.

M. Quetelet has formed Tables of Mortality for Belgium, shewing the number dying every year out of 10,000 born, deduced from the deaths of males and females in town and country; but, as these tables take no account of the living, which, instead of remaining fixed, were ever changing, they are open to the objections before stated that stamp all such tables with inutility. M. Quetelet's tables can no more be considered Tables of Mortality for Belgium, than the tables calculated by Dr. Lombard, M. Heyer, and Mr. Rickman, are Tables of Mortality for Geneva and Essex: he has besides neglected to give the proportions living and dying at each age, and of each sex, in town and country, from which true tables of mortality may be formed.

In the assurance offices of this country many millions of pounds sterling depend on the duration of human life, and on the law of mortality. The transactions in which this money is concerned are carried on by two parties; the assurers, proprietors of offices perfectly acquainted with the numerical and commercial details of the question, and the public, who as a body know less of the law of mortality than of almost any other matter in which they are directly interested. The consequences have been that assurance companies, with little *certain* capital, speculated and levied enormous profits on the public hope and imagined foresight, which, unguided by knowledge, became ruinous credulity. Many of them divided the contributions, which from the construction of their tables, accumulate in the first years; and, in no long period, were found more mortal than the lives they promised to assure. Confiding families were overwhelmed with ruin. Many of the present assurance societies deserve the entire confidence of the assured: it is not, however, our intention to discuss their respective merits; we only wish to notice their tables of mortality, made on what they call the *safer* side. If, out of one hundred persons assured, at the age of forty, *twenty* died before the age of fifty, speculators ought to pay for the chance of having 1000*l.* at death, occurring during that period, 200*l*; for there are twenty chances in favour of their receiving 1000*l.*, and eighty chances against their receiving it: on the contrary, if only ten in one hundred die in the same time, the deposit ought only to amount to 100*l.*, for it is ninety to ten that they live, and that the 1000*l.* are lost.* Again, if from the fortieth year of life, men on an average live twenty-eight years, to receive 1000*l.* at death they ought to pay a smaller annual sum than if the average duration of after-life was only twenty-three years. These examples shew that it is advantageous for assurance offices to employ tables underrating the probability and duration of human life; accordingly, they apply the numbers of the Northampton Table, professing only to shew the mortality of an entire population, poor and rich, and representing *that* much higher than was ever observed: they apply, we repeat, this *safe* table to determine the mortality of men in the middle classes, holding the most durable tenure of life; and, besides this, exclude all the sick class, out of which the greater part of the mortality indicated in their table necessarily takes place. If, in the English population at the age of 40—50, fifteen in one thousand die, the one thousand include among them at least thirty constantly sick; out of whom the deaths in the tables generally take place: therefore, by excluding the sick, and by means of a medical certificate,† the mortality among the assured falls at first immeasurably short of that indicated by the tables of mortality. From the tables published by the Equitable Office, it appears that, while among those *selected* at thirty, the mortality between forty-five and fifty years was one in fifty-eight, the mortality at the same age of those who were *selected* at forty-five, was only one in ninety-two; a vast difference in favour of the office.

These facts in no way prove that the assurance of life is injudicious; we signalize them as glaring misapplications of the known principles of life admeasurement: a general class of men indiscriminately taken, healthy and sick, are presumed to indicate the mortality of another class from which the *sick* and *feeble* are excluded.

* No account is here taken of the rate of interest, which is taken at 3 per cent.; as the lower the interest of money is assumed to be, the greater are the profits of the assurance offices.

† Before granting a policy, the assurance offices address a schedule to some medical practitioner, requesting his confidential opinion on the state of the applicant's health, &c. Of late there has been some controversy on the question, whether the Directors, by whom professional opinions of this kind are demanded, should pay the accustomed fee; and, in the eyes of all, except one of our contemporaries, it has appeared evident that merely *mercantile* bodies have no right to obtain a deliberate, written, professional opinion, gratuitously. Let us add, that the Offices, by means of this opinion, and the selection they thus exercise, realise an enormous profit. According to their tables and rates, a person aged 40, to insure for £100, should live for 23 years, and pay 3*l.* 8*s.* annually. Now, by means of the medical opinion, and the selection, the assurance offices only grant policies to persons who live and pay, on an average, 28 years. So that, if a person insures his life for £5000, they gain nearly £1000 more than is sufficient to afford a fair remuneration on their capital. It, therefore, does appear to us fair, that they should pay the medical fee. In truth, the assurance offices have taken advantage of the good-nature, and, we must confess, of the ignorance of the medical profession: but we hope the many honorable men connected with these institutions will no longer attempt to perpetuate an imposition which the medical profession is bound, in justice to itself and the public, to resist.

In the case of annuities, the selection is in favour of the annuitants; and, as the proportion of sick increases up to old age, the difference at this period becomes very great between the deaths out of 1,000 taken indiscriminately from the general population, and one thousand from which thirty or forty per cent. of sick and ailing have been excluded. Through inattention to this fact, the nation recently lost several hundred thousand pounds by granting annuities on the lives of old men and women, selected by speculators on the Stock Exchange.

---

